# Communication skills of medical students during the OSCE: Gender-specific differences in a longitudinal trend study

**DOI:** 10.1186/s12909-017-0913-4

**Published:** 2017-05-02

**Authors:** Joachim Graf, Robert Smolka, Elisabeth Simoes, Stephan Zipfel, Florian Junne, Friederike Holderried, Annette Wosnik, Anne M. Doherty, Karina Menzel, Anne Herrmann-Werner

**Affiliations:** 1 Medical Faculty Tuebingen, Dean’s Office for Students’ Affairs, Geissweg 5/1, D-72076 Tuebingen, Germany; 2 Department of Women’s Health, Research Institute for Women’s Health, University Hospital Tuebingen, Calwerstrasse 7, D-72076 Tuebingen, Germany; 3 Department of Women’s Health, University Hospital Tuebingen, Calwerstrasse 7, D-72076 Tuebingen, Germany; 4 DRK Clinic Center Berlin, Hospital for Psychosomatic Medicine and Psychotherapy, Spandauer Damm 130, D-14050 Berlin, Germany; 5 Staff Section Social Medicine, University Hospital Tuebingen, Hoppe-Seyler-Strasse 6, D-72076 Tuebingen, Germany; 6 Department of Psychosomatic Medicine and Psychotherapy, University Hospital Tuebingen, Internal Medicine, Osianderstrasse 5, D-72076 Tuebingen, Germany; 7 Department of Gastroenterology, Hepatology and Infectious Diseases, University Hospital Tuebingen, Internal Medicine, Otfried-Müller-Strasse 10, D-72076 Tübingen, Germany; 8 Department of Psychiatry, University Hospital Galway, Galway, Ireland; 9 Section of Public Health, University Hospital Duesseldorf, Moorenstrasse 5, D-40225 Duesseldorf, Germany; 10 Interdisciplinary Training Centre DocLab, Medical Faculty Tuebingen, Elfriede-Aulhorn-Strasse 10, D-72076 Tuebingen, Germany

**Keywords:** Communications skills, Medical students, Self- and external perception, Gender differences, Osce, Gender-specific teaching

## Abstract

**Background:**

Communication skills are essential in a patient-centred health service and therefore in medical teaching. Although significant differences in communication behaviour of male and female students are known, gender differences in the performance of students are still under-reported. The aim of this study was to analyse gender differences in communication skills of medical students in the context of an OSCE exam (OSCE = Objective Structured Clinical Examination).

**Methods:**

In a longitudinal trend study based on seven semester-cohorts, it was analysed if there are gender differences in medical students’ communication skills. The students (self-perception) and standardized patients (SP) (external perception) were asked to rate the communication skills using uniform questionnaires. Statistical analysis was performed by using frequency analyses and t-tests in SPSS 21.

**Results:**

Across all ratings in the self- and the external perception, there was a significant gender difference in favour of female students performing better in the dimensions of empathy, structure, verbal expression and non-verbal expression. The results of male students deteriorated across all dimensions in the external perception between 2011 and 2014.

**Discussion & conclusion:**

It is important to consider if gender-specific teaching should be developed, considering the reported differences between female and male students.

## Background

### Communication skills of medical students

In order to be a ‘good doctor’, physicians require not only clinical and scientific knowledge, but also excellent communication skills to ensure a good doctor-patient-relationship [1–6]. Good communication skills in physicians can be understood as a multidimensional phenomenon, which is characterised by an emphasis on patients’ expectations, concerns and emotions and their need for information. Relationship building, negotiating and facilitating patients’ co-operation are also core elements of communication skills [7]. As highly developed communication skills are crucial for doctor-patient interactions, it is recommended to incorporate them in teaching from the very beginning at medical school [8, 9]. In Germany, the medical faculties are faced with the challenge of implementing more practical examinations instead of written and oral exams to promote the social, communication and interpersonal skills in the teaching [10, 11]. Empathy and other aspects of communication skills of medical students are often in a need of optimization, but it is known that they can be improved by training programs in the context of medical teaching [12–15]. The development of standardized patients (SP) may be especially useful in improving the communication performance of the students [16]. Many medical schools introduced the OSCE (Objective Structured Clinical Examination) to examine communication skills, a meaningful and recognized way to improve the general doctor-patient communication [17, 18]. With the OSCE, it is possible to consider the students social, communication and interpersonal skills, which have been taught in special courses previously [17, 18]. The OSCE exam is a circuit of brief examinations, in which the students must demonstrate their communication skills and practical abilities by completing different tasks at each station, including history talking or physical examinations involving SP where required in different medical specialities [19–23].

### Communication skills: Gender specific aspects

It is well known that communication styles of female physicians interacting with patients are consistently different from their male counterparts: female doctors ask more psychosocial questions, receive more positive patient talk, and demonstrate more positive nonverbal communication [24–26]. Female physicians typically show more empathy and use more positive statements than males when interacting with their patients [27]. When communicating diagnosis-specific information, male general practitioners (GPs) use more instrumental behaviour (giving information), while female GPs use more affective behaviour (giving attention, reassurance) [28, 29]. Better communication competences in female students are found than in male students: it has been shown that female students score higher than their male counterparts after a training course in communication skills [30]. Other studies show that female students obtain higher empathy scores than male students [31, 32]. In the OSCE exam, female students have significantly better results in the communicative sections than their male counterparts [33] and show a better performance in most of the stations [34]. Female students are more sensitive in the doctor-patient relationship, but feel significantly less confident than male students in the OSCE [35]. However, it remains unclear whether the gender differences in communication performance in the OSCE could be the result of SP’s gender, because male and female SPs may sometimes differ in how they rate examinees overall [26, 36]. Wiskin demonstrated that there is no significant relationship between SP gender and the result of the student, while the examiner’s gender apparently affected the results: Male examiners assess the communication skills of the female students significantly better than the female examiners, while both examiners gender rates female students significantly better than men [34]. Other studies demonstrate a significant influence of the SP gender: male students performed worse when interacting with male SP, and all students performed better when interacting with the female counterparts [37]. Although significant differences in the communication behaviour of male and female physicians are reported in studies, gender aspects are rarely taken into account in medical teaching of communication skills [28]. Despite the relevance of communication skills in medical teaching, gender differences in the performance of the students in the OSCE are still underreported.

In the light of competency-based developments like the CanMeds framework [38] or the German National Competence-Based Learning Objectives Catalogue [39], where the physician’s role as communicator is explicitly acknowledged and valued, it is crucial to identify students’ needs – not only in general, but also with regards to gender differences. To our knowledge, studies existing so far only used one-dimensional approaches (e.g. only assessors’ view). However, as, due to their nature, external ratings are highly subjective, only one perspective might not be enough. Consequently, we decided to approach our research question in a multidimensional way by comparing external perception (SP) with students’ self-perception. To our knowledge, there are no longitudinal studies, in which the multidimensional phenomenon of students’ communication skills is analysed in a gender-specific approach across all dimensions of communication. Thus, the aim of this study was to analyse gender differences in communication skills of medical students in the context of an OSCE exam in a longitudinal approach in relation to the fact that the perspective (self- vs. external) influences the perception and so determines the response behaviour in the questionnaires.

## Methods

The design was conceived as a longitudinal trend study. Trend studies (also called replicative surveys) represent the third subtype of longitudinal analyses (in addition to cohort and panel studies). A trend study samples different groups of people at different points in time but in the same situation and from the same population. The aim is to demonstrate the development of skills or attitudes in social groups like medical students, whereby not the individual, but the whole group gets focalized. While in cohort studies *the same* persons are interviewed at regular intervals (e.g. the same medical students in the course of studies, therefore in the first, second, and the other semesters), trend studies pursue the target to survey *different persons of the same population* at regular intervals (e.g. the students of the sixth semester in an OSCE looking at several consecutive OSCEs every half-year). So, trend studies use cross-sections at two or more points in time to examine change over time within a population [40–42]. This trend study based on seven semester-cohorts, examining the communication skills of medical students from Tuebingen University at the end of the 6th semester to identify gender differences in the communication performance of the students. Before participating in the OSCE, students were asked to rate their own communication skills (self-perception). During the OSCE all SP were asked to rate the students’ communication skills (external perception). So the trend study was designed as a full-survey, because we interviewed all SP and all students performing the OSCE exam each semester. Both groups completed standardized uniform questionnaires to rate the following four dimensions of communication: empathy, structure, verbal expression and non-verbal expression, using a five- and six-point Likert scale, respectively. On the self-perception scale, 1 reflected “completely disagree” and 6 “completely agree”. In the external rating of skills, 1 reflected the worst performance and 5 the best (see Tables 1 and 2). Our medical students are familiar with completing such questionnaires and required no training. The SP completed a standardized training based on video case studies to enable competent assessment. Many SP are professional actors with diploma. In Tuebingen, SP have been a crucial part of medical training since 2004. Their operations are coordinated by a central programme with a designated quality management scheme. Basic SP training follows international standards [43–45] and is additionally modified by students’ evaluation to constantly monitor and improve performance. All SP involved in the OSCE have longstanding experience in medical teaching sessions. Summed up, the SP used in this study are thus qualified to assess the students’ communicative performance. In this study, we analysed the four dimensions of communication as rated by students (self-perception) and SP (external perception) and compared them to identify possible differences between female and male students’ performance in the four dimensions: empathy, structure, verbal expression and non-verbal expression. We also analysed gender-specific differences between self- and external perception of the communication skills. Additionally, we analysed any changes over the semesters in the different cohorts. For data processing, Microsoft Excel 2010 and IBM SPSS 21 were used. First we carried out a frequency analysis in order to identify the descriptive characteristics (means and related distributions (SD)) of the data. Subsequently, we conducted unpaired t-tests for independent samples. The data were normally distributed in both dimensions (self- vs. external perception). In all analyses, a *p*-value of <0.05 was considered to be statistically significant (α = 0.05). We used the Bonferroni correction to counteract the problem of multiple comparisons. Where there was homogeneity of variances on Levene’s test, we performed an ANOVA. When there was no homogeneity, we performed an unpaired t-test between the first and the last cohort. Before we analysed the gender-specific differences in self- und external perception, reliability and validity of both questionnaires were examined by performing multiple correlation, Cronbach’s Alpha, item-total correlation, inter-item correlation and factor analyses.

**Table 1:** Items of the self-perception rating (students)

Item: Right now, I feel able to…..	Rating
1.) … answer sympathetically to the verbal and non-verbal cues and needs of my counterpart (empathy)	1 = completely disagree; 2 = rather disagree; 3 = partly accept; 4 = rather agree; 5 = agree; 6 = completely agree
2.) … organize a conversation coherently and direct the flow of the conversation (structure)	
3.) … adapt my manner to my counterpart in wording, voice modulation, speech rate etc. (verbal expression)	
4.) … motivate my counterpart in the conversation by using non-verbal techniques (non-verbal expression)

**Table 2:** Items of the external perception rating (standardized patients)

Item	1 2 3 4 5	Item
1.) The student does not respond to the obvious (verbal and nonverbal) cues and needs from me as a SP and/or responds inappropriately (empathy)		1.) The student always responds to the obvious (verbal and nonverbal) cues and needs from me as a SP and/or responds appropriately (empathy)
2.) The conversation is not organized recognizably; the student acts incoherently or I as SP have to set the course of the conversation (structure)		2.) The conversation is excellently organized. The student’s approach shows, that the (s)he is able to direct the conversation (structure).
3.) The student communicates inappropriately with me as a SP (e.g. choice of words, volume) and/or communicates in a way, that makes it impossible to understand him (verbal expression)		3.) The student communicates appropriately with me as a SP (e.g. choice of words, volume) and/or communicates in a way, that makes it easy for me to understand him (verbal expression)
4.) The student does not manage to involve me as SP with his non-verbal expression and frustrates me and/or antagonizes me (non-verbal expression).		4.) The student successfully involves me as a SP in the communication with his non-verbal expression and/or motivates me to participate (non-verbal expression)

## Results

### Student population: Socio-demographic characteristics

One thousand twenty seven students from 7 semester cohorts (summer semester 2011 to summer semester 2014) were recruited. The average age of students across all 7 cohorts was 24.9 ± 3.85 years. The gender distribution of the total student population was 60% female and 40% male (for further details see Table 3). Both questionnaires (the 6-point Likert-skaled self-perception and the 5-point Likert-skaled external perception assessment) were reliable and valid, because Cronbach’s Alpha and the item-total correlation amount to >0.7 and the factor analyses were >0.8 (see Table 4).

**Table 3:** Characteristics of the student population: age and gender

Semester	Number of students	Gender distribution		Age: Mean (Range (Min; Max)) [SD]
		Male	Female	
2011	162	32% (*n* = 52)	68% (*n* = 110)	24.86 (27 (21;48)) [4.19]
2011–2012	168	42% (*n* = 71)	58% (*n* = 97)	25.40 (38 (21;59)) [4.24]
2012	148	34% (*n* = 51)	66% (*n* = 97)	24.38 (32 (20;52)) [3.61]
2012–2013	81	44% (*n* = 36)	56% (*n* = 45)	25.93 (25 (21;46)) [4.66]
2013	150	40% (*n* = 60)	60% (*n* = 90)	24.75 (13 (21;34)) [3.03]
2013–2014	165	47% (*n* = 77)	53% (*n* = 88)	25.29 (33 (19;52)) [3.98]
2014	153	41% (*n* = 63)	59% (*n* = 90)	23.7 (19 (20;39) [2.93]
Total	1027	40% (*n* = 410)	60% (*n* = 617)	24.84 (24) [3.85]

**Table 4:** Reliability and validity analyses of self perception- and external perception dimensions

	Multiple correlation	Cronbachs Alpha	Item-total correlation	Inter-item-correlation [95%-CI]	Factor analyses
Self-perception rating					
Empathy	0.705	0.848	0.834	0.690 [0.622; 0.767]	0.913
Structure	0.572	0.878	0.752		0.861
Verbal expression	0.646	0.865	0.786		0.884
Non-verbal expression	0.558	0.886	0.728		0.845
External perception rating					
Empathy	0.592	0.860	0.763	0.674 [0.625; 0.719]	0.871
Structure	0.518	0.876	0.719		0.839
Verbal expression	0.604	0.854	0.776		0.879
Non-verbal expression	0.629	0.849	0.788		0.888

### Self-perception of communication skills: gender specific differences

In general, students of both gender rated their communication skills in all dimensions as good. When analysing the total of all seven semester cohorts, female students rated themselves better than their male counterparts across all four dimensions of communication **(**Table 5). The largest gender-specific differences in the rating concerned the dimensions of empathy (mean female = 4.46; mean male = 4.25) and non-verbal expression (mean female = 4.15; mean male = 4.06), while slight differences exist in the dimensions of structure and verbal expression. In the total sample, female students rated their skills in the dimension of empathy highest (average score 4.46), closely followed by the dimensions of verbal expression (mean = 4.42) and structure (mean = 4.39), while the non-verbal expression was assessed less positively (mean = 4.15). In the male students, other priorities were found: here, the dimension of verbal expression was rated highest (average score 4.40), followed by the dimension of structure (mean = 4.37). In contrast, the skills of the males in relation to empathy and non-verbal expression were assessed less positively (mean = 4.25 and mean = 4.06). Because the female students rated themselves better than their male counterparts in all four dimensions, we analysed whether the differences between males and females were statistically significant. A significant association was found in the dimension of empathy (*p* = 0.0039), while the differences in the other three dimensions in favour of female students were not statistically significant (Table 6). Comparing the mean scores (self-perception) of the first and last semester cohort (summer semester 2011 versus summer semester 2014), in both genders the dimensions of structure and non-verbal expression improved, while the verbal expression was rated lower in 2014 than in 2011. The dimension of empathy scored better only in the male cohort, while the same dimension remained unchanged in the females (Table 7). The changes in the self-perception were not statistically significant between 2011 and 2014. We could not perform ANOVA, because there was no homogeneity of variances on Levene’s test.

**Table 5:**
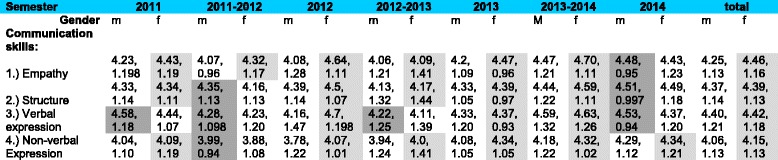
Gender-specific analysis of the self-perception rating across the four dimensions

Mean *, Standard Deviation; light grey = female students (f) performed better, dark grey = male students (m) performed better. * for all Items in all dimensions: range = 5 (Min = 1; Max = 6)

**Table 6:** Statistical analysis of gender-specific differences in the self-perception rating of communication skills

	Male (*n* = 410)		Female (*n* = 617)		Difference	95%-CI	*p*-value (α = 0.05)
	Mean	SD	Mean	SD			
Empathy	4.25	1.13	4.46	1.16	−0.21	−0.35; −0.07	**0**.**0039**
Structure	4.37	1.14	4.39	1.13	−0.02	−0.16; −0.12	0.7641
Verbal expression	4.40	1.21	4.42	1.18	−0.02	−0.17; 0.13	0.7864
Non-verbal expression	4.06	1.13	4.15	1.13	−0.09	−0.23; 0.05	0.2309

**Table 7:** Change of the self-perception between 2011 and 2014 (unpaired t-test) in male and female students

Male students	t1 = 2011 (*n* = 52)		t2 = 2014 (*n* = 63)		Difference	95%-CI	*p*-value (α = 0.05)
	Main	SD	Main	SD			
Empathy	4.23	1.198	4.48	0.95	0.25	−0.65; 0.15	0.2146
Structure	4.33	1.14	4.51	0.997	0.18	−0.57; 0.21	0.3684
Verbal expression	4.58	1.18	4.53	0.94	−0.05	−0.34; 0.44	0.8008
Non-verbal Expression	4.04	1.10	4.29	1.12	0.25	−0.66; 0.16	0.2323
Female students	t1 = 2011 (*n* = 110)		t2 = 2014 (*n* = 90)		Difference	95%-CI	*p*-value (α = 0.05)
	Main	SD	Main	SD			
Empathy	4.43	1.19	4.43	1.23	0.0	−0.34; 0.34	1.0
Structure	4.34	1.11	4.49	1.18	0.15	−0.47; 0.17	0.3565
Verbal expression	4.44	1.07	4.37	1.20	−0.07	−0.25; 0.39	0.6635
Non-verbal Expression	4.09	1.19	4.34	1.21	0.25	−0.59; 0.09	0.1440

### External perception by standardized patients

The external perception of communication skills as rated by the SP was different from the self-perception ratings of the students. In total *n* = 8484 communication sheets were analysed: each student (*n* = 1027) was assessed on average by 8.24 SPs. In the external perception, the female students were rated better than their male counterparts across all four dimensions of communication over all semesters. Here the highest rate was in the dimension of verbal expression (mean = 4.33), followed by the dimension of empathy (mean = 4.298). In the dimension of non-verbal expression the female students were rated with an average score of 4.23, with lowest scores in the dimension of structure (mean = 4.15). The male students showed the same pattern in the external perception: the dimension of verbal expression was rated highest (mean = 4.25), followed by empathy (mean = 4.22), non-verbal expression (mean = 4.15) and structure (mean = 4.10) **(**Table 8).

**Table 8:**
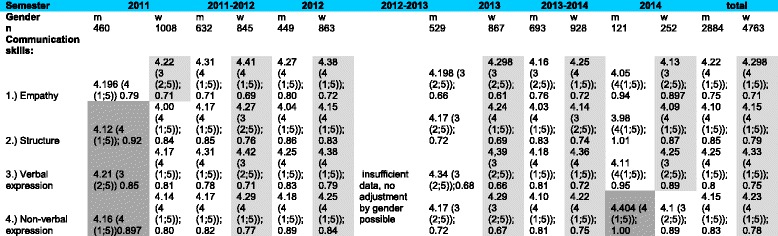
gender-specific analysis of the external perception dimensions

Mean (Range (Min; Max)), Standard Deviation; light grey = female students (f) performed better, dark grey = male students (m) performed better

The same gender-specific mean differences were found in the dimensions of empathy (mean female = 4.3; mean male = 4.22; difference: 0.08; CI: -0.1138/ -0.0465, *p* = <0.0001), verbal expression (mean female = 4.33; mean male = 4.25; difference: 0.08; CI: -0.1186/ -0.0471, *p* = <0.0001) and non-verbal expression (mean female = 4.23; mean male = 4.15; difference: 0.08; CI: -0.1137/ -0.0397, *p* = <0.0001), while the differences in the dimension of structure were less (mean female = 4.15; mean male = 4.10; difference = 0.05; CI: -0.0878/ -0.127, *p* = 0.0085). In all four dimensions of communication, female students performed better than their male counterparts, and gender differences were statistically significant (Table 9).

**Table 9:** statistical analysis of the gender-specific differences in the external perception

	Male (*n* = 2884)		Female (*n* = 4763)		Difference	95%-CI	*p*-value (α = 0,05)
	Mean	SD	Mean	SD			
Empathy	4.22	0.75	4.3	0.71	−0.08	−0.1138; −0.0465	**<0**.**0001**
Structure	4.10	0.85	4.15	0.79	−0.05	−0.0878; −0.0127	**0**.**0085**
Verbal expression	4.25	0.8	4.33	0.75	−0.08	−0.1186; −0.0471	**<0**.**0001**
Non-verbal expression	4.15	0.83	4.23	0.78	−0.08	−0.1137; −0.0397	**<0**.**0001**

Comparing the mean scores (external perception) of the first and last semester cohort (summer semester 2011 versus summer semester 2014), the SP ratings of the communications skills in females and males developed very differently. In all four dimensions, the SP rated the male students in 2014 worse than in 2011. In the dimension of non-verbal expression, we found a statistically significant worsening (*p* = 0.0096) between the first (summer semester 2011) and the last cohort (summer semester 2014). While the external perception of all four skills in the males deteriorated over the timeline, the communication skills of the female students showed a mixed picture. The SP rated the females’ competences in the dimensions of structure and verbal expression better in 2014 than in the summer semester 2011, whereas the dimensions of empathy and non-verbal expression worsened over the time. In all dimensions, the differences between the first and the last cohort in female students were much lower than in the males: we did not find statistically significant effects in the females (Table 10).

**Table 10:** Change of the external perception between cohorts examined in 2011 and 2014 (unpaired t-test) in male and female students

Male students	t1 = 2011 (*n* = 460)		t2 = 2014 (*n* = 121)		Difference	95%-CI	*p*-value (α = 0.05)
	Main	SD	Main	SD			
Empathy	4.196	0.79	4.05	0.94	−0.15	−0.019; 0.31	0.0832
Structure	4.12	0.92	3.98	1.01	−0.14	−0.05; 0.33	0.1452
Verbal expression	4.21	0.85	4.11	0.95	−0.10	−0.08; 0.28	0.2620
Non-verbal Expression	4.16	0.897	4.404	1.00	−0.24	−0.43; −0.06	**0.0096**
Female students	t1 = 2011 (*n* = 1008)		t2 = 2014 (*n* = 252)		Difference	95%-CI	*p*-value (α = 0.05)
	Main	SD	Main	SD			
Empathy	4.22	0.71	4.13	0.897	−0.09	−0.014; 0.19	0.0891
Structure	4.00	0.84	4.09	0.87	0.09	−0.21; 0.03	0.1312
Verbal expression	4.17	0.81	4.25	0.89	0.08	−0.19; 0.03	0.1696
Non-verbal Expression	4.14	0.80	4.10	0.89	−0.04	−0.07; 0.15	0.4880

## Discussion

Overall, the communication skills of students in the dimensions of empathy, structure, verbal expression and non-verbal expression can be described as acceptable as measured in this OSCE, when rated by students themselves and SP. But it remains open, how the results can be transferred to other exam situations or even further into practise. There is a lack of transferability studies, which analyse the correlation between the communicative results of an OSCE exam and the later performance in practise. Nevertheless, we found gender differences in all four dimensions of self- and external perception, and demonstrated that female students performed better in all dimensions of the analysed communication skills. In the whole collective, women were better than men in all four dimensions of communication (empathy, structure, verbal expression and non-verbal expression) in both rating perspectives (self- and external perception). In general, ratings by SP was better than the students one: mean scores were 4.10–4.25 in men, and 4.15–4.33 in women on a 5-point Likert-scale, whereas mean scores in self-evaluation on a 6-point Likert-scale were 4.06–4.37 in male students, and 4.15–4.46 in female students, respectively. Interestingly, in male students a differing trend in self- and external perception could be seen by focalising differences between 2011 and 2014: there was an improvement in 3 of 4 dimensions in the self-perception, while external perception worsened. This phenomenon was described as overestimating one’s clinical and communicational competences in literature and can be found more often in male students [46]. Although our results are consistent with other studies showing that female students obtain higher empathy scores than men [31, 32], no previous studies report such gender differences across all four dimensions of communication described above (empathy, structure, verbal expression, non-verbal expression). Despite the fact, that gender is a well-known variable in the assessment of communication skills [34], and although gender effects determine medical communication [24, 25, 47], to our knowledge no study so far has been looking at the various of communication from two perspectives with a focus on gender differences. We demonstrated that female students are outperforming their male counterparts in communication skills as rated both by the self- and the external perception, and the differences in the SP ratings in favour of female students were statistically significant. The influence of the SP gender is negligible, because the same number of male and female SP were used during the OSCE: each student was assessed by equal proportions of male and female SP. Nevertheless, there could be a limitating factor regarding the standardized patients as we only collected their gender but no further data – particularly no pseudonym or code. Thus, it was impossible to link any rating to the corresponding SP and calculate Fleiss` Kappa and the inter-rater reliability. An interesting finding is that the male students not only performed poorly compared to the females, but that the cohorts examined also worsened over time in all dimensions of the external dimension, while the females showed a lesser negative change in only two dimensions. This may be related to the decrease in age of medical students: in summer semester 2011 the average age of students was 24.86 year; by summer semester 2014 it had decreased to 23.7 years. Due to the conversion to an 8-year secondary school system and an abolition of a mandatory military and civil service, students in Germany (especially male students) are now much younger at enrolment in university than a few years ago. The improvement in empathy scores in male students between 2011 and 2014 is also an interesting finding. We demonstrated that the male performance on the empathy scale improved on self-perception, but worsened on external perception. This is confirmed by other studies that demonstrated lower empathy values but higher self-confidence rates in male students [35], but also shows that male students are more unrealistic in estimating their communication competences in empathy than females. Overall, the study confirms that gender specific aspects of medical education are neglected [28]. Gender medicine teaching is still in the early stages in Germany, but it is clear that it has to implement in the health care and also in the medical teaching [48–51]. There is a need for a paradigm shift in medical care, research and teaching, to reach both genders similarly and to counteract existing gender-specific stereotypes [49]. In the art of conversation, men and women in general as well as female and male physicians in particular [24–29] differ from each other, rendering it is necessary to integrate gender aspects in the teaching of communication skills, to increase the competences especially of the male students. The results of the study sustain the demand for implementing gender-specific teaching formats for improving students’ communication skills. Within these, well-known gender-specific reservations towards doctor-patient communication and its teaching should be addressed [52–55]. However, the aim should not be to eliminate differences in the way in which men and women communicate, but rather to look at inequality as an opportunity to improve the competences of male and female students individually. There is great relevance in improving communication skills in medical students especially in the context of existing gender-specific differences in order to improve the physician-patient interaction for enhancement of patient care [2, 3, 5]. Especially the tendency of male students for overestimating their own clinical and communicative skills can pose a danger to patient’s safety [46], wherefore it is necessary to optimize their skills. Finally it could be demonstrate that the subjectivity of surveys for measurement of communication skills plays an increasingly important role, because there were differences between self- and external perception in the semester-overall analyses. This suggests a possibly existing bias in other studies especially in those with a focus on only one dimension of perception. Using a multidimensional approach might be broadening this picture. Further studies could look into the clinical transfer, or the assessors as a second external source of perception could additionally be included in the analyses.

## Conclusions

Medical students in Tuebingen showed good overall communication skills in the four dimensions of empathy, content structure, verbal expression and non-verbal expression, with gender-specific differences in all dimensions in favour of female students. With male students underperforming in all dimensions, the development of additional gender-specific teaching should be considered.

## Abbreviations

GP: General Practitioners ; OSCE: Objective Structured Clinical Examination ; SP: Standardized Patients
